# Social dynamics modeling of chrono-nutrition

**DOI:** 10.1371/journal.pcbi.1006714

**Published:** 2019-01-30

**Authors:** Alessandro Di Stefano, Marialisa Scatà, Supreeta Vijayakumar, Claudio Angione, Aurelio La Corte, Pietro Liò

**Affiliations:** 1 Dipartimento di Ingegneria Elettrica, Elettronica e Informatica (DIEEI), CNIT (National Inter-University Consortium for Telecommunications) Catania, Italy; 2 Department of Computer Science and Information Systems, Teesside University, Middlesbrough, United Kingdom; 3 Computer Laboratory, University of Cambridge, Cambridge, United Kingdom; National Cancer Institute, United States of America and Tel Aviv University, Israel, UNITED STATES

## Abstract

Gut microbiota and human relationships are strictly connected to each other. What we eat reflects our body-mind connection and synchronizes with people around us. However, how this impacts on gut microbiota and, conversely, how gut bacteria influence our dietary behaviors has not been explored yet. To quantify the complex dynamics of this interplay between gut and human behaviors we explore the “gut-human behavior axis” and its evolutionary dynamics in a real-world scenario represented by the social multiplex network. We consider a dual type of similarity, homophily and gut similarity, other than psychological and unconscious biases. We analyze the dynamics of social and gut microbial communities, quantifying the impact of human behaviors on diets and gut microbial composition and, backwards, through a control mechanism. Meal timing mechanisms and “chrono-nutrition” play a crucial role in feeding behaviors, along with the quality and quantity of food intake. Considering a population of shift workers, we explore the dynamic interplay between their eating behaviors and gut microbiota, modeling the social dynamics of chrono-nutrition in a multiplex network. Our findings allow us to quantify the relation between human behaviors and gut microbiota through the methodological introduction of gut metabolic modeling and statistical estimators, able to capture their dynamic interplay. Moreover, we find that the timing of gut microbial communities is slower than social interactions and shift-working, and the impact of shift-working on the dynamics of chrono-nutrition is a fluctuation of strategies with a major propensity for defection (e.g. high-fat meals). A deeper understanding of the relation between gut microbiota and the dietary behavioral patterns, by embedding also the related social aspects, allows improving the overall knowledge about metabolic models and their implications for human health, opening the possibility to design promising social therapeutic dietary interventions.

## Author summary

Human gut microbiota is able to influence different aspects of physiology, such as human behaviors. Our close social connections, in turn, impact on eating behaviors and diets, which play a key role in driving the gut microbial composition and its metabolic processes. To quantify the dynamic interplay between gut microbiota and human behaviors in a social multiplex network, we investigate the “gut-human behavior axis”. We propose statistical estimators able to quantify the relation between human behaviors and gut microbiota in a social multiplex network. Furthermore, considering a population of shift workers, we explore the dynamic interplay between their eating behaviors and their gut microbiota, quantifying the social dynamics of chrono-nutrition in a multiplex network. Our findings, coherently with what one can expect in the case of shift-working, demonstrate how the timing of gut microbial communities is slower than social interactions and shift-working. Thus, the impact of shift-working on chrono-nutrition is a fluctuation of behaviors, with a major propensity for poor food choices or high-fat meals. Acquiring a deeper knowledge about the metabolic models of gut microbiota and quantifying the complex interplay of social and gut microbial communities pave the way to targeted social therapeutic interventions.

## Introduction

Recently, the human gut microbiota has emerged as a major research topic in many fields, from biology to neuroscience and medicine [1–5]. One of the main targets is to find out and explain how it may influence different aspects of physiology, such as gut-brain communication and human behaviors [1, 6–8]. Recent research has highlighted the role of the commensal gut microbiota in brain function and behavior, revealing a bidirectional communication between gut and brain, known as gut-brain axis [2, 8]. Some further works have shed light on the pivotal role of diet and other environmental factors in driving the composition of the human gut microbiota and its metabolic processes that, in turn, can affect human health [9, 10]. Many studies have also stressed the importance of gut microbiota and its alterations in the emergence of various diseases, including metabolic disorders, low-grade inflammations, type 2 diabetes, neurodegenerative diseases and obesity [2, 11–17].

Diet is considered one of the most important factors impacting on the human gut microbiota [9, 10]. As a result, many research efforts have been focusing on applying dietary interventions to better understand mechanisms linking diet, gut microbiota, diet-induced behaviors and mental health [11, 15]. Moreover, the importance of evaluating the diet-induced dynamics of the human gut microbiota has been underlined, both in terms of individuals and groups or clusters of people [10], including their heterogeneous and individual nature [18]. Gut microbes compete for nutrients and this in turn will affect metabolism, making the gut microbiota an extremely dynamic metabolic organ associated with host health and disease phenotypes [19]. The long-term stability of gut microbial communities, along with the responsiveness to physiological change, confirms the potential of the gut microbiota as a diagnostic tool and therapeutic target [20].

Dietary behaviors and patterns depend on a wide variety of aspects, such as educational and familiar habits, geographical location, psychological factors, including mood, impulsiveness and physiological conditions [6, 21]. Nutritional factors act in concert with inherited factors—through epigenetic mechanisms—to increase an individual’s risk for developing impulsive, compulsive and conduct disorders which are present in general population to various degrees [22]. The influence of gut microbiota on behavior is becoming increasingly evident [1]. Therefore, the evidence that nutritional habits modulate gut microbiota composition and function offers new opportunities for the prevention and treatment of behavioral disorders [23].

Reasoning in terms of social networks, the influence of social interactions on our dietary choices can result in a convergence of behaviors and a diet synchronization with those of our close social connections [24]. This creates ‘rewarding clusters’ that may act as resonant cavities amplifying and reinforcing some behaviors. These clusters are a clue of how social context is crucial to understand how and why people with whom we share meals can influence what we eat. To model this behavior in our work, the formation of clusters is obtained according to a metabolic measure based on gut bacteria similarity (see Materials and methods), which also reflects the affinity and homogeneity of bacteria [25, 26]. The involvement in one or more clusters and the interactions through social ties within groups can produce either positive (e.g. happiness) or negative feelings (e.g. stress) [27]. These conditions are often the result of unconscious biases [28], reflecting an attitude or a bias triggered by our brain, which is out of our awareness and control, influencing our choices. In our work, we include the two concepts of micro-inequities and micro-affirmations acting in opposite directions, representing two manifestations of this bias [28]. Since chronic stress facilitates the inflammatory action of bacteria [29], this suggests the importance of these psychological biases in influencing nodes’ or clusters’ behaviors. As well as considering the gut similarity, we also include the concept of homophily, namely the principle for which similarity breeds connection [30–32], as a similarity measure depending on interactions between nodes. Then, in order to model and quantify the mutual influences between gut microbiota and the social multiplexity of networked individuals in the “gut-human behavior axis”, we introduce a feedback mechanism, acting from dietary behaviors influenced by social interactions to gut microbiota and, backwards, from gut to social behaviors.

Given the evidence that food intake and dietary behaviors are partially influenced by the social relationships and contexts, multiplex networks constitute the most suitable and natural description for analyzing these dietary behaviors in a real-world scenario such as social networks. Multiplexity allows capturing the role of different types of relationships among the same set of nodes, including several layers, or networks, and characterizing the complex nature of interactions [18, 30, 31, 33, 34]. Additionally, multiplex networks can be used to simulate adaptation to environmental conditions (such as changes in diet or differential gene expression) by integrating multiple data types (‘omic’ layers) using network aggregation [35]. Here, in order to obtain a quantitative measure of the change in metabolic flux in response to various dietary regimes, a bidirectional interaction is recorded between a multiplex network of social ties and a genome-scale metabolic model (GSMM), which provides a reconstruction of all biochemical reactions taking place within a multi-species microbial community of the human gut. This presents the opportunity to monitor the impact of macronutrient intake on an individual’s health and to evaluate the possibilities for improving their health through dietary intervention. In the context of the gut microbiome [5], metabolic modeling holds the potential to capture the complex interplay between social relationships and metabolic profiles, as the flux distribution predicted under each condition is subject to the feedback from the social network as well as the dietary composition of each individual.

Along with the quality and the quantity of food intake, feeding time becomes crucial in eating habits and impacts on metabolic and physiological parameters, with possible dramatic effects on health outcomes [36–40]. Recent research efforts have highlighted how the rotation between day and night leads to a high-calorie diet, an excessive energy intake (sugars, glucose, etc.) and dysregulated dietary habits (e.g. consumption of snacks or junk food) [36, 38, 41]. This contributes to the desynchronization of the circadian system and the subsequent development of diseases, such as metabolic syndrome, cardiovascular diseases, obesity and depression [40, 42–48]. This is a condition typically experimented by shift workers [36, 40, 41, 43, 49], with recent studies highlighting their impaired glucose regulation and changes in metabolism [50, 51]. These changes may produce several consequences, such as increase the risk of type 2 diabetes and obesity in the long-term [36, 37, 42–44, 46, 52]. Shift workers tend to eat foods with a high energy intake to stay awake during the shifts, e.g. caffeine and high sugar foods; these become part of their diets as they provide quick boosts of energy in order to improve alertness during their shifts [36, 43, 53, 54].

Meal timing and patterns are linked to the concept of “chrono-nutrition”, which refers to food intake in coordination with the body’s daily rhythms, shedding light on the importance of temporal eating patterns on the well-being of an organism [37, 38, 40, 55, 56]. Thus, chrono-nutrition focuses on how our dietary habits influence our biological clock and circadian rhythm [42, 55, 56]. Circadian rhythms allow us to exploit the energy intake from foods, but shift-working may alter these rhythms in a 24-hour period. Likewise, diet dynamics change the gut microbiota nearly in the same period [9]. Some other works have also underlined the critical role played by the gut microbiota in the clock-nutrition interplay [37, 57], so that the disruption of the circadian physiology, due to sleep disturbances or shift-working, may result in gastrointestinal diseases, such as Irritable Bowel Syndrome (IBS), an increased risk of obesity, diabetes, cardiovascular disease, and cancer [36, 38, 40, 43, 44, 50]. In fact, chrono-disruption influences circadian rhythms and hence the gut-brain axis [58], contributing to the pathogenesis of several diseases of the digestive system [40].

In our model, we therefore include shift-working in order to quantify the impact of chrono-nutrition. This choice is further justified by the definition of “health” provided by the World Health Organization (WHO), where “shift work” is a risky condition at various levels, since it is not only a risk factor for many health disorders (e.g. gastrointestinal, psychoneurotic, cardiovascular, reproductive functions, and probably cancer), but it also perturbs psycho-physical homeostasis (e.g. sleep/wake cycle and circadian rhythms) and hinders family and social life [45, 46, 49]. In order to understand the complex dynamics of dietary behaviors of shift workers, we need to quantify the dynamic interplay between human behavior and gut microbiota by exploiting the introduced social and psychological statistical estimators. Only by reasoning in terms of social multiplex network, we are able to evaluate the multiple impact of different domains or channels of interactions between individuals, including the social environment (e.g. workplace and friends), where shift workers are included, which may be able to influence and modify their feeding habits acting on focus groups (e.g. by introducing a food-sharing scheme in the workplace) [49].

**Fig 1:**
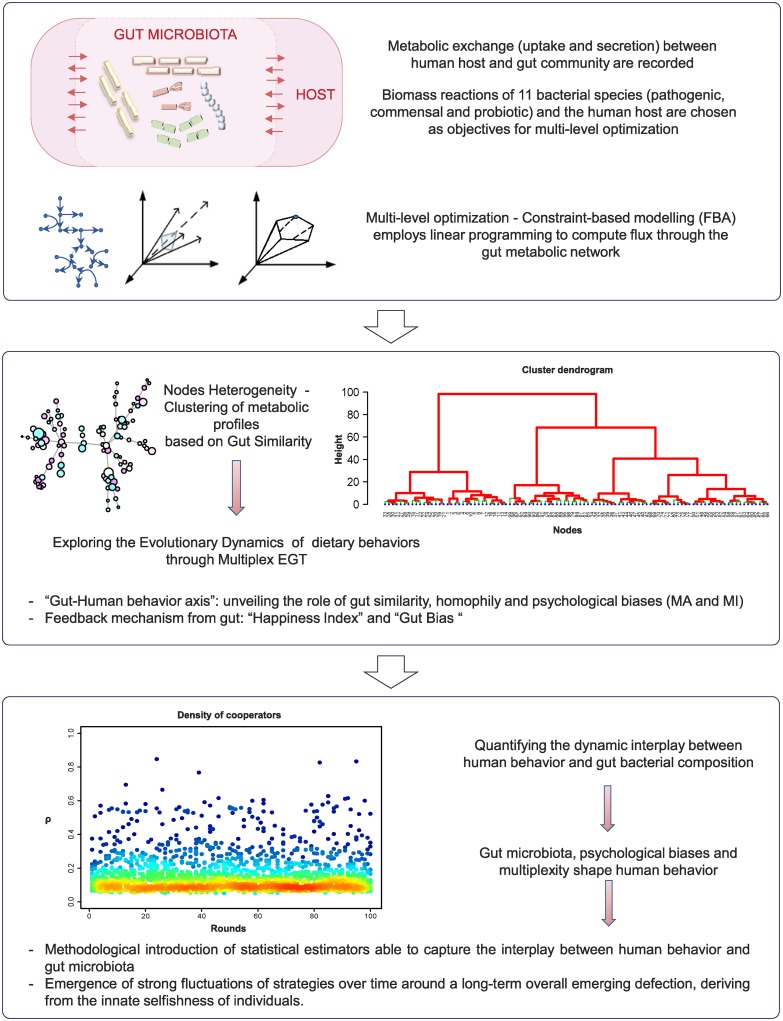
Modeling approach. Bio-molecular step (top panel); associations between humans and gut bacterial species, clustering based on gut similarity (middle panel); dynamic interplay between human behavior and gut microbiota (bottom panel).

Our work aims at quantifying the bidirectional interplay between dietary behaviors emerging from the social evolutionary dynamics of feeding habits and the gut microbial composition. To explore and quantify this interplay on a social multiplex network, we exploit the framework of Evolutionary Game Theory (EGT) [30, 31], which is used to simulate the effect of dietary perturbations on each node of the multiplex network. We quantify the evolutionary social dynamics of chrono-nutrition by considering the interplay between human behavior and gut microbiota. Analyzing the formation of social and microbial communities based on gut-similarity, as well as homophily and psychological biases, may suggest how to implement methods and strategies able to reduce metabolic disorders. To the best of our knowledge, this work represents the first attempt to join social and metabolic issues in modeling the social dynamics of chrono-nutrition, exploring the “gut-human behavior axis” in a multiplex network and quantifying their dynamic interplay through the introduction of statistical estimators (see Fig 1). In Table 1 we briefly describe each step of our modeling procedure.

**Table 1:** Steps of the proposed modeling procedure. We list and briefly describe the various steps of our modeling procedure, indicating the references to the figures and subsections of the section Materials and methods.

**Fig 2:**
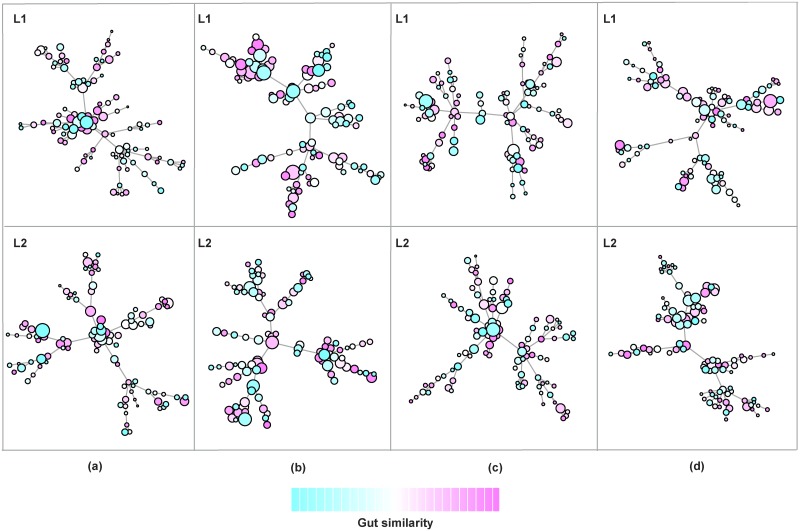
Structural and metabolic heterogeneity of nodes—Gut similarity and structural properties of nodes in the multiplex network. We show the heterogeneity of nodes in terms of diets and gut similarity (*θ*_*ij*_) among nodes in the different layers of the multiplex network (*L*_1_ and *L*_2_), along with their structural measure of centrality and homophily [30]. The color, ranging from ‘light cyan’ to ‘violet’ passing through ‘white’, allows characterizing the distance among nodes in terms of gut similarity, so that the more similar are diets and gut bacterial composition, the closer will be the colors in the plot. The node size is log-proportional to the overall measure of centrality and homophily in the multiplex network [30]. For each column of the panel, we show the gut similarity before feedback (a), after the first feedback round (b), after the second feedback round(c), and after the third feedback round (d).

**Fig 3:**
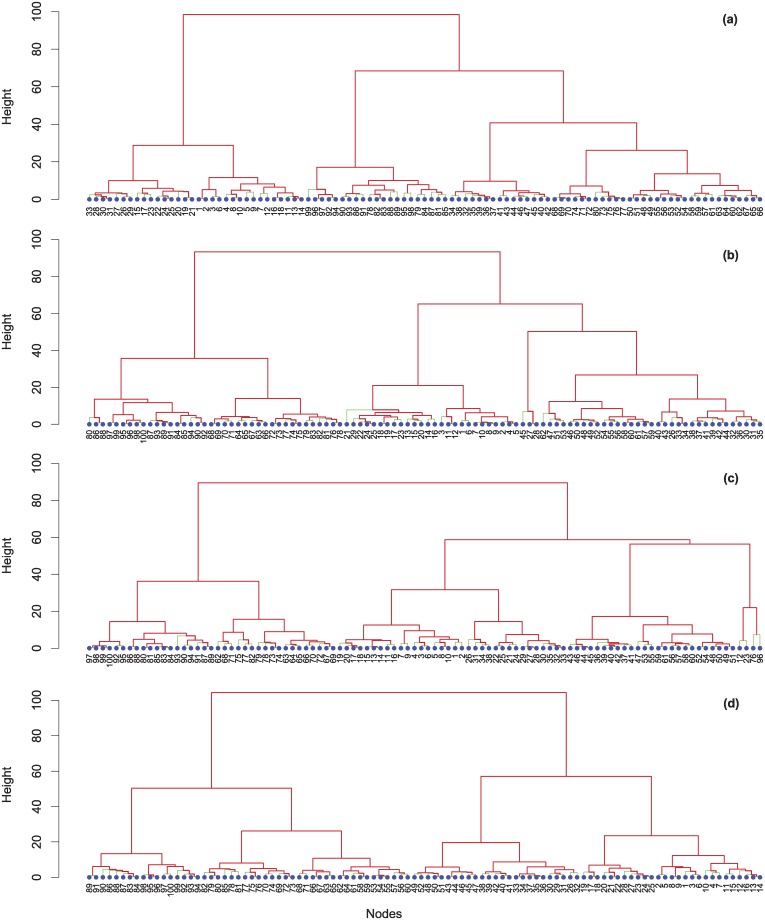
Hierarchical clustering of nodes in the multiplex network based on gut similarity. We show the hierarchical clustering and re-clustering of nodes based on their gut similarity in the multiplex network in the various temporal windows: before feedback (a), after the first feedback round (b), after the second feedback round (c), after the third feedback round (d). Nodes change the clusters to which they belong according to their modifications in dietary uptake rates.

Step and Description
1. Initialization of the multiplex network (*N* = 100 nodes, *M* = 2 layers), diets and gut profiles, and “happiness index” (*γ*_*i*_ = 0) (see Evolutionary dynamics on a social multiplex network and Feedback mechanism)
2. Evaluation of “gut similarity” (*θ*_*ij*_) and initial hierarchical clustering (see Figs 2 and 3a)
3. ISG is played and strategies are updated at each round of the game following Eq 1) (see Evolutionary dynamics on a social multiplex network and Micro-affirmations and micro-inequities)
4. Diets and gut profiles are updated based on the feedback mechanism (see Feedback mechanism and Metabolic model)
5. Simulation of the metabolic gut model (FBA) with new constraints derived from step 4 (see Metabolic model)
6. Re-clustering based on the updated value of “gut similarity” (see Metabolic model and Fig 3b–3d)
7. Steps 3-6 are repeated

## Materials and methods

### Evolutionary dynamics on a social multiplex network

Let us consider a multiplex network of *M* layers, *α* = {1, …, *M*}, and *N* nodes, *i* = {1, …, *N*}, which is a set of *M* networks *G*_*α*_ = (*V*, *E*_*α*_). The set of nodes *V* is the same for each layer, whereas the set of links *E* changes according to the layer.

Each layer corresponds to a different kind of social interaction between nodes of the multiplex network and it may exhibit a different topology of connections. Modeling a social network as a multiplex network represents a more realistic approach, since in most real-world social networks, different and not mutually exclusive relationships can be considered between the same individuals (e.g. friends, relatives, colleagues, etc). Thus, the framework of social multiplex network allows us to analyse the complex dynamic patterns involving these individuals, shedding light on how the different types of interactions at various layers impact on the realistic dynamics of human behaviors.

We start from the key assumption that each entity or node in the multiplex network is an individual with a different gut metabolic profile, which in turn reflects its specific diet (see Metabolic model). Each network *G*_*α*_ is described by its adjacency matrix $A^{\alpha }\in R^{N\times N}$, where each element is given by: $a_{ij}^{\alpha }=1$, if nodes *i* and *j* are connected at layer *α*, or $a_{ij}^{\alpha }=0$ otherwise.

The idea is to define clusters of individuals based on the concept of “gut similarity”, a metabolic similarity measure related to their metabolic profiles and indicated by *θ*_*ij*_, calculated as an Euclidean distance between metabolic profiles. Since the metabolic profile is linked to diet, nodes with similar dietary habits will belong to the same cluster. Nodes are the same in every layer of the multiplex network, so they will belong to the same cluster in all the layers of the multiplex network. In terms of homophily, we consider an overall measure that includes both the concepts of centrality and homophily in the multiplex structure [30]. Moreover, we weigh the coupling between layers in the multiplex exploiting the communicability function defined in [30], which quantifies the number of possible routes that two nodes have to communicate with each other. In order to analyze the emergent behaviors in the clusters and in the whole multiplex, we consider the mathematical framework of EGT on multiplex networks [30, 31]. In particular, we choose the Snowdrift Game (SG), a pairwise social dilemma [59, 60] where nodes can select one of the two strategies, cooperation or defection. The symmetric game can be described with the payoff matrix in Table 2.

**Table 2:** Payoff matrix of the Snowdrift Game. In this social dilemma, a task has to be done and *b* is the benefit of accomplishing the task, while *c* is the cost for doing the task (where $b,c\in R_{+}$ and *b* > *c*). We can observe that if neither of the two players puts an effort to cooperate, the task would not be accomplished with a negative effect for both players.

	Cooperate	Defect
**Payoff to Cooperation**	*b* − *c*/2	*b* − *c*
**Payoff to Defection**	*b*	0

In this model of conflict, a task has to be done and defectors benefit (*b*) from cooperators without paying a cost (*c*) for doing the task, but without cooperators the task would not be accomplished. Thus, despite mutual cooperation yields both an individual and total benefit higher than that of mutual defection, pure defection is the favored strategy when the other player cooperates, which occurs at the cost of the overall group payoff. It is clear how both strategies are best replies to each other, which leads to a “coexistence game” [61]. In our model, the choice of cooperating, namely following a healthy lifestyle and diet and sharing dietary habits with neighborhood, can derive from the direct and indirect reciprocity mechanisms [62]. Indeed, good eating habits may positively influence the overall behavior of the group a node interacts with, by stimulating the other nodes to be more proactive and producing, as a consequence, an amplifying effect. Instead, the selfish choice of defecting, leveraging the cooperativeness of the other neighboring nodes, without paying any cost of cooperating, can produce a major payoff in the short time, but it constitutes a major risk if also the other players will decide to defect.

One of the main reasons behind the choice of an Iterated Snowdrift Game (ISG) in this scenario is linked with its more realistic nature compared to other social dilemmas [59]. Indeed, it corresponds to more frequently observed natural situations where cooperators contribute to a public good or common task, represented in our model by good dietary habits, that is exploitable by cheaters but also provides immediate direct benefit to the cooperator [59]. In evolutionary games, payoffs determine reproductive fitness, and the target is to detect the Evolutionarily Stable Strategy (ESS). This can be formalized using replicator dynamics [30, 31] which, in the case of SG, admits a mixed stable equilibrium, where both cooperation and defection coexist. The pairwise nature of the game is translated to a population scale by making the nodes play with each other, and accumulating payoffs obtained from each interaction, over the rounds of the game. Thus, we consider an ISG where, after each round of the game, the strategies of the nodes are updated so that those nodes with less payoff are inclined to imitate the strategy of the fittest individuals. We focus on memory-1 game [63], so that choices at each round depend only on the previous round. In [63] the authors have proved that, giving only a finite memory of previous play, the payoff obtained is exactly the same as if we would consider a player with a longer memory.

We simulate the evolutionary microscopic dynamics using the standard Monte Carlo simulation procedure, composed of elementary steps, where each step gives a chance for every player to change its strategy once on average. The first step includes the distribution of competing strategies, which is an elementary step entails randomly selecting a player and one of its neighbors, calculating the payoffs of both players, and finally attempting a strategy adoption. First, a randomly selected player *i* acquires its payoff *P*_*i*_ by playing the game with all its neighbors on the layer *α*. Next, player *i* randomly chooses one neighbor *j* on the layer *β*, who then also acquires its payoff *P*_*j*_ on the layer *β* in the same way as previously did player *i*. Thus, a player *j* imposes its strategy *S*_*j*_ onto player *i* with a probability determined by the Fermi function, defined as follows [30]:

$$\begin{array}{c} W\left(S_{j}\to S_{i}\right)=\left(\eta_{i}\right)\cdot \left(\psi_{i}\right)\cdot \frac{1}{1+exp[\left(P_{i}-P_{j}\right)/\left(\theta_{ij}\cdot \delta_{ij}\cdot K\right)]}. \end{array}$$ (1)

Therefore, a player *i* adopts the strategy *S*_*j*_ of another player *j* in function of the payoff difference *P*_*i*_ − *P*_*j*_, the two similarity measures (*δ*_*ij*_ and *θ*_*ij*_) and the factors *η*_*i*_ and *ψ*_*i*_, related to multiplexity and unconscious bias, respectively. In particular, we consider the homophily measure, *δ*_*ij*_, related to social interactions, and the gut similarity measure, *θ*_*ij*_, related to gut metabolic profiles. Moreover, we include two statistical estimators *η*_*i*_ and *ψ*_*i*_ in the multiplex network, where *η*_*i*_ is a factor related to communicability measure in the multiplex network [30], while *ψ*_*i*_ represents the balance of micro-affirmations (*MA*) and micro-inequities (*MI*) of each node in the multiplex (see Micro-affirmations and micro-inequities). The selection intensity *K* quantifies the uncertainty in the strategy adoption process [30]. The homophily measure *δ*_*ij*_ represents the homophily difference between two players, so that if this values is high, a player *i* is more likely to imitate the strategy of *j* at each round.

The scaling factor *η*_*i*_ allows including the dependency of the strategy adopted by the player *i* on the strategies of related players from the other layers [30], so it depends on the communicability function between layers [30, 64]:

$$\begin{array}{c} \eta_{i}=1-\left(\eta_{i_{max}}-\eta_{i_{min}}\right)\frac{\sum_{j\in \beta ,S_{j}=S_{i}}{\left[G_{\alpha \beta }\right]}_{ij}}{\sum_{j\in \beta }{\left[G_{\alpha \beta }\right]}_{ij}}, \end{array}$$ (2)

where the numerator is the sum of the communicability functions calculated between the node *i* on the layer *α* and all the neighboring nodes *j* on the layer *β*, adopting the same strategy as player *i*. The denominator represents the sum of the communicability functions calculated between the node *i* on the layer *α* and all the neighboring nodes *j* belonging to the layer *β*. Therefore, the ratio quantifies the influence, in terms of communicability, on the strategy adoption of the player *i* on the layer *α*, due to the strategies adopted by the counterpart node and its neighbors on the layer *β*. In particular, the more are players on the layer *β* with a high communicability with the node *i* and adopting the same strategy as player *i*, the more likely *i* will adopt the same strategy in the next round. On the other hand, if there are nodes on the layer *β* with a high communicability, but adopting a different strategy, the player *i* will most likely tend to change its strategy. Thus, this ratio depends on the communicability function and it may result in a bias regarding the strategy adoption of the player *i* in the next round of the game.

The communicability function has been introduced in order to shed light on the importance of the coupling between layers in exploring the evolution of behaviors in the multiplex structure. To this aim we have exploited the communicability function defined in [64], which quantifies the number of possible routes that two nodes have to communicate with each other. Starting from the definition of the communicability function between two nodes *i* and *j* in the multiplex provided in [64], we consider the communicability matrix *G*, where each element $G_{\alpha \beta }\in R^{N\times N}$ is the matrix representing the communicability between every pair of nodes belonging to two different layers *α* and *β*, of the multiplex network. It is defined as in [30] and [*G*_*αβ*_]_*ij*_ represents the communicability between the node *i* in the layer *α* and the node *j* in the layer *β*.

### Micro-affirmations and micro-inequities

In order to deal with unconscious bias, in our model we introduce a statistical estimator *ψ*_*i*_, impacting on Eq 1. This bias, which is the result of quick judgments or evaluations of people and situations, is influenced by our individual experiences or environment in which we are merged, occurs automatically and may impact on our choices [28]. “Micro-inequities” (*MI*) are one of the manifestations of this bias, and they are defined as “apparently small events which are often ephemeral and hard-to-prove, events which are covert, often unintentional, frequently unrecognized by the perpetrator, which occur wherever people are perceived to be different” [28]. In order to address it, Rowe defined the concept of micro-affirmations (*MA*) as “apparently small acts, which are often ephemeral and hard-to-see, events that are public and private, often unconscious but very effective, which occur wherever people wish to help others to succeed” [28]. Micro-affirmations therefore act as a countermeasure to micro-inequities, so that if individuals are constantly proactive about rewarding and professing the achievements of others, people will be less inclined to micro-inequities and it will trigger a reciprocity effect, producing a psychological reward.

It is important to observe how the unconscious bias, expressed in terms of *MI* and *MA* and quantified by the factor *ψ*_*i*_ (see Eq 3), is linked with several environmental (cultural, geographical, social, etc.) factors, metabolic needs and emotional states [21], influencing our food choices and the resulting gut microbial composition [65, 66]. *ψ*_*i*_ is defined as follows:

$$\begin{array}{c} \psi_{i}=1-\left(\psi_{i_{max}}-\psi_{i_{min}}\right)[1-\sum_{j}\frac{MI_{ji}}{MA_{ji}+MI_{ji}}], \end{array}$$ (3)

where *MA*_*ji*_ and *MI*_*ji*_ are respectively the micro-affirmations and micro-inequities obtained at each round of the game by the node *i* from all the interactions with its neighboring nodes *j*. At each round, the factor *ψ*_*i*_ is calculated in game-theoretical terms, by taking into account all the payoffs obtained by the node *i* in the previous round of the game. In particular, if a node gets a “positive” payoff (see the first column of the payoff matrix, Table 2), then it will get a *MA*, otherwise a *MI*. Thus, *ψ*_*i*_ produces a bias in the choices and, in particular if *MI*_*ji*_ ≥ *MA*_*ji*_, then a player will have a higher tendency to change its strategy in the next round. It may reflect a sort of ‘delusion’ or ‘sadness’ of the node *i*, related to the payoff obtained in the previous round after having played with its neighboring nodes *j*. Otherwise, if *MI*_*ji*_ ≤ *MA*_*ji*_, it means that node *i* has experienced positive affirmations in its interactions (“positive” payoff) and it will likely tend to keep the same choice adopted in the previous round. Analogously to *η*_*i*_ [30], the two limit values, $\psi_{i_{max}}=1$ and $\psi_{i_{min}}=0.1$, are chosen in order to avoid the “frozen states”. *ψ*_*i*_ acts as a key bias towards a change in strategy when *MI*_*ji*_ ≫ *MA*_*ji*_, which means a strong imbalance of *MI* over *MA*, while if *MA*_*ji*_ ≫ *MI*_*ji*_, a node tends to keep the same strategy of the previous round. At the beginning we assume that *MI*_*ji*_ and *MA*_*ji*_ are initialized with a value equal to zero, which creates an initial condition where nodes are on average satisfied of their neighborhood and diet, given that there have not been interactions and hence they have not yet experienced any *MA* or *MI* in the network.

### Feedback mechanism

Our work is aimed at modeling and quantifying the mutual influences between gut microbiota and the social multiplexity of networked individuals in the “gut-human behavior axis”. To this end, we introduce a control mechanism, acting from dietary behaviors influenced by social interactions to gut microbiota and, backwards, from gut to social behaviors. Although the diet-induced dynamics of the gut microbial communities has a relatively high responsiveness within 24 hours [9], diets and bacterial composition of gut microbiota are determined by long-term dietary habits [10]. Thus, we evaluate the evolutionary dynamics in a relatively long time-span, including a certain amount of rounds of the game (see Results).

To measure the impact of these long-term dietary behaviors on diets and human gut microbiota, we introduce a statistical estimator *γ*_*i*_, defined as follows:

$$\begin{array}{c} \gamma_{i}=\sum_{i}{\left(NC\right)}_{i|t-1}/N_{r}, \end{array}$$ (4)

where *NC* is the number of cooperations in the previous *t* − 1 rounds before feedback, while *N*_*r*_ is the number of rounds before feedback, which happens at time step *t*. *γ*_*i*_, called “happiness index”, reflects the attitude of each individual in the network, that is how much a node has been “happy” (or cooperative) in the previous rounds. Therefore, *γ*_*i*_ will act as a modulator, influencing gut metabolic profiles and modifying a fraction of diet components (nutrients, etc.), and ultimately producing a different gut profile. *γ*_*i*_ ranges in [0, 1], where *γ*_*i*_ = 0 reflects a lack of cooperativeness while *γ*_*i*_ = 1 means that a node has been fully cooperative with a proactive attitude towards its community. *γ*_*i*_ quantifies the impact on gut bacteria deriving from human behaviors. The updated gut metabolic model for each individual will then be used to update the distance among individuals for the next round, therefore driving the re-clustering of the multiplex network based on the gut similarity of the updated gut profiles (see Metabolic model for details on how a different diet composition is achieved and used to run te gut model, finally updating the gut flux profile of each individual).

As well as producing a re-clustering of the nodes in the multiplex network, *γ*_*i*_ can produce a remarkable *MA* or *MI*, that is respectively a positive or negative bias from gut. This bias, weighted by another statistical estimator *ϵ*_*i*_, called “gut bias”, is defined as the ratio between the number of *MA* or *MI* obtained in all the rounds before the feedback mechanism and the number of rounds in the same temporal window. We have a dual definition of *ϵ*_*i*_, depending on the sign of the bias from gut, so that in the case of a positive bias, it is defined as:

$$\begin{array}{c} \epsilon_{i}=\sum_{i}MA_{i|t-1}/N_{r}, \end{array}$$ (5)

whereas in the case of a negative bias it is defined as:

$$\begin{array}{c} \epsilon_{i}=\sum_{i}MI_{i|t-1}/N_{r}. \end{array}$$ (6)

In terms of evolutionary dynamics, the “gut bias” amplifies the number of *MA* or *MI*, impacting on *ψ*_*i*_ and, in turn, on the strategy adoption process (see Eq 1). In particular, we define a threshold *γ*_*th*_ so that, if *γ*_*i*_ ≥ *γ*_*th*_, it means that the node has experienced a positive feedback, resulting in: *MA* = *MA* + *ϵ*_*i*_ ⋅ *MA*, while if *γ*_*i*_ < *γ*_*th*_, the node has experienced a negative feedback and there is an amplifying effect of *MI* as follows: *MI* = *MI* + *ϵ*_*i*_ ⋅ *MI*. The re-clustering of the network, obtained at each control mechanism, modifies the spatial distribution of the network. It is interesting to observe the patterns of clustering depending on the evolutionary dynamics of nodes behaviors, due to the described feedback mechanism.

### Metabolic model

The happiness index *γ*_*i*_ is used to update the diet of each individual during each feedback round. The impact of the new diet on the gut microbiota is assessed using a GSMM. GSMMs can be used for generating context-specific or personalized gut microbiota models through nutrient composition or data integration at various omic levels, and have recently been augmented with three-dimensional metabolite and protein structure data [67–70]. The model used to represent the gut consists of a human GSMM (Recon 2) and reconstructions for eleven bacterial species representing commensal, probiotic and pathogenic associations which can occur between the human host and gut microbiome [71]. Flux balance analysis (FBA) is then used to run the updated model. FBA is characterized by the definition of precise linear constraints used to cumulatively reduce the solution space of the computed states of a metabolic model. After selecting a cellular objective, the model is finally simulated through linear programming [72].

For a genome-scale metabolic model representing a single organism or community, biomass production is the most commonly considered objective for optimization as it can serve as a proxy for growth rate. To improve this estimation, in recent years procedures have been developed for checking biomass weight, generating accurate stoichiometric coefficients for the biomass objective function and enforcing time-averaged equality of growth rates in microbial communities [73–75]. For this combination of models we use the COBRA Toolbox in MATLAB [76] to perform FBA whilst optimizing a linear combination of multiple biomass objectives, which represents hierarchical maximization of biomass production by each member of the microbial community (see Table 3).

**Table 3:** Ordered list of biomass reactions chosen as objectives for multi-level linear optimization in FBA for the different species considered in this study.

Species	Abbreviation	Biomass reaction name	Objective
*Bacteroides thetaiotaomicron*	BT	BTBiomass_BT_v2	c_1_
*Faecalibacterium prausnitzii*	FP	FPBiomass_FP	c_2_
*Escherichia coli* K-12 MG1655	EC	ECEC_BIOMASS_IAF1260_WT_59P81M	c_3_
*Lactobacillus plantarum*	LP	LPbiomass_LPL60	c_4_
*Lactococcus lactis*	LL	LLbiomass_LLA	c_5_
*Streptococcus thermophilus*	ST	STbiomass_STR	c_6_
*Helicobacter pylori*	HP	HPBiomassHP_published	c_7_
*Klebsiella pneumoniae*	KP	KPBiomass	c_8_
*Salmonella typhimurium*	STy	STybiomass_iRR1083	c_9_
*Escherichia coli* O157:H7 (Sakai)	ECs	ECsEC_BIOMASS_IAF1260_WT_59P81M	c_10_
*Escherichia coli* O157:H7 (EDL933)	ECe	ECeEC_BIOMASS_IAF1260_WT_59P81M	c_11_
*Homo sapiens* Recon 2	HS	HSbiomass_reaction	c_12_

In our modeling framework, a series of 100 diets with varying compositions of carbohydrate, fat and protein intake was created to simulate the effect of various diets on metabolic flux through the optimized pathway(s) or reaction(s). With the exception of a few more extreme diets, percentages of macro-nutrients within these diets were generated using a random number generator. Subsequently, uptake rates were calculated by multiplying percentages of each macro-nutrient by a basal uptake rate *B*, thus accounting for differences in molecular size by scaling uptakes to the number of carbon atoms in each nutrient [71].

Following Heinken and Thiele [71], 53 metabolites belonging to three classes of macronutrients—carbohydrates, fats and proteins (*C*, *F*, and *P*) were associated with a basal uptake rate *B* calculated from a data survey conducted by the USDA [77]. These basal uptake rates were multiplied by the specific percentage of each macronutrient in an individual’s diet. With the exception of the Western diet (94.36% sugars, 5.64% fiber), carbohydrate composition for all diets was divided into 50% sugars and 50% fiber. Therefore, basal uptake rates for fats and proteins were multiplied by 2 to re-scale them in accordance with the carbohydrate uptake. All of the diets contain the same number of metabolites, but uptake rates were varied by adjusting lower bounds for the exchange reactions of each metabolite with respect to the type of diet simulated. For example, the calculation of metabolite uptake rates (*A*_*u*_) for a diet consisting of 34% carbohydrate, 44% fat and 22% protein would be calculated as follows:

$$\begin{array}{c} \begin{array}{cc} C_{u} & =0.34\cdot B_{C}\\ F_{u} & =0.44\cdot 2B_{F}\\ P_{u} & =0.22\cdot 2B_{P}\\ A_{u} & =[C_{u},F_{u},P_{u}]. \end{array} \end{array}$$ (7)

Series of uptake rates were calculated separately for each group of nutrients (i.e. *C*_*u*_, *F*_*u*_ and *P*_*u*_) before being concatenated within a single vector of uptake rates (*A*_*u*_). Once uptake rates for all metabolites were calculated, these values were set as lower bounds of exchange reactions in the model using indexes corresponding to each reaction. In this way, FBA returns a complete flux distribution for each diet, elucidating beneficial, detrimental or neutral effects of dietary changes on human health.

When this modeling approach is combined with social relationships using a hierarchical clustering based on gut profiles, the complex interplay between dietary habits and social relationships can be analyzed by examining interactions between nodes representing metabolic profiles and social ties. In modeling diets, we must take into account some perturbations of diets as people do not adhere to dietary regimes, corresponding to the most typical diets (e.g. Western diet, high-fat, high-carbohydrate, high-protein), but most often they change some dietary components producing diet modifications or perturbations, that will result in a change in the microbiota [10].

We jointly optimize the growth of all species (including the host) by setting biomass reactions for each species as objectives using a multi-level linear optimization program based on GEMsplice [78] which seeks trade-offs between multiple objectives. The order of the chosen biomass objectives was defined as: BT, FP, EC, LP, LL, ST, HP, KP, STy, ECs, ECe and HS [79] (see Table 3). The growth of commensal and probiotic species were considered to be more important as they are thought to produce beneficial metabolites for the host as opposed to toxic or harmful compounds produced by the pathogenic species. In accordance with [71], a minimal growth rate was enforced to maintain growth of all species by constraining the lower bounds for the chosen biomass objectives to 0.01. To set this, we define v_biomass_ as all of the biomass reactions within the full flux vector *v* (i.e. those reactions identified by the objectives *c*_1_, …, *c*_12_ and detailed in Table 3). For each diet, fluxes through the biomass and ATP synthase reaction for each species were recorded. In each distribution, fluxes for the biomass and ATP synthase reaction are used to calculate new values for *γ*_*i*_, which are averaged over all previous rounds of EGT. Formally, we defined the twelve-level linear program as follows:

$$\begin{array}{c} \begin{array}{cccccccc} & \text{max}\; & & c_{12}\cdot v\\ & \text{s}.\;\text{t}.\; & & \text{max}\;c_{11}\cdot v,\\ & & & \ldots \\ & & & \text{s}.\;\text{t}.\; & & \text{max}\;c_{2}\cdot v,\\ & & & & & \text{s}.\;\text{t}.\; & & \text{max}\;c_{1}\cdot v,\\ & & & & & & & \text{s}.\;\text{t}.\;\;\;\Lambda v=\dot{x}\\ & & & & & & & \;\;\;v^{\text{min}}\leq v\leq v^{\text{max}}\\ & & & & & & & \;\;\;v{}_{\text{biomass}}^{\text{min}}=0.01\\ & & & & & & & \;\;\;{\dot{x}}_{j}=0,\text{if}\;M_{j}\in \text{internal}\;\text{metabolites}\\ & & & & & & & \;\;\;{\dot{x}}_{j}\in R,\text{if}\;M_{j}\in \text{external}\;\text{metabolites}, \end{array} \end{array}$$ (8)

where Λ is the stoichiometric matrix of the model, *x*_*j*_ represents the concentration of each metabolite *M*_*j*_.

Upon obtaining the first set of *γ*_*i*_ values for every node (characterized by a specific diet) in the network, the initial percentage of each macronutrient (carbohydrate, fat and protein) ±(1 − *γ*_*i*_) ⋅ 10 is used to calculate lower and upper limits, between which the new fraction of each dietary component is selected for that node using a random number generator for the next feedback, ensuring that the sum of all three percentages is equal to 100%. For example, if a given node in the first feedback round has a carbohydrate percentage of 50 and the *γ*_*i*_ value for this diet characterizing the node *i* is 0.0800586, we calculate: (1 − *γ*) ⋅ 10 = (1 − 0.0800586) ⋅ 10 = 9.19941. This means that in the next feedback round, the lower carbohydrate limit will be equal to: 50 − 9.19941 = 40.80059, and the upper carbohydrate limit will be: 50 + 9.19941 = 59.19941. Therefore, for this particular node *i*, the carbohydrate percentage in next feedback round is set to a randomly drawn integer between 40.80059 and 59.19941, e.g. 54%. The calculation is repeated using the same *γ*_*i*_ for adjusting fat and protein content in this node *i*, and using a different *γ*_*i*_ value for each node *i* in all four feedback rounds.

The gut community model used for this study comprises multiple bacterial species within the human host. There are also particular gut communities, that is those formed by patients with celiac disease [80], autistic individuals [81] or people with irritable bowel syndrome [82]. In [83], an inter-organ communication during metabolic disorders was identified, and in fact almost every sick organ guides the formation of a community. Although some studies consider a larger number of bacterial species, in [14] the number of species considered is similar. Here the cause of community formation is the disease, but it may also be the therapy administration (e.g. metformin for type 2 diabetes). We can observe how specific diseases, geographical locations or even cultural choices (e.g. vegan diets) link the community and gut in a stronger way than other situations.

Following [35, 84], the metabolic gut flux profiles of each individual are then used to define distances among individuals. This metabolic similarity, represented by *θ*_*ij*_ and calculated as an Euclidean distance between metabolic profiles is then used to define clusters of individuals.

## Results

In our model, we aim at evaluating the combined role of gut similarity, micro-affirmations, micro-inequities and multiplexity in shaping the strategies of an individual within a community. Simulations were conducted choosing a multiplex network with *M* = 2 layers, where each layer is modeled as a scale-free network [85, 86] with *N* = 100 nodes. Each node corresponds to an individual following a specific diet calculated by randomly perturbing the percentage composition of macronutrients. Homophily values are randomly chosen following a normal distribution around a mean value, with standard deviation *σ* [30]. Fig 2 shows the gut similarity among nodes in the multiplex network, along with their structural measure of centrality and homophily [30]. This picture of the multiplex network allows us to depict the heterogeneity in terms of diets and composition of gut. Each node reacts in a different way to social interactions and feedback rounds from gut, producing a different behavior. Gut similarity rules the formation of clusters and then impacts on the choices of nodes, together with the various feedback rounds from their own gut.

Fig 3 illustrates the hierarchical clustering of nodes in the multiplex network based on their gut similarity in the different temporal windows before feedback and after each feedback round from gut. We have used an agglomerative hierarchical clustering based on the Ward method [87], where the dissimilarities are squared Euclidean distances between cluster means. Ward method refers to the sum of the squared distance from each point to the mean of the merged clusters, where the distance between two clusters is the sum of the squared deviations from points to centroids. Thus, we use the Euclidean distance between metabolic profiles as a distance metric. More specifically, the dissimilarity structure is in our case the Euclidean distance between metabolic compositions of gut microbiota, so that our clustering is then performed based on this measure of distance. Then, the Ward clustering method based on minimum variance allows us to perform agglomerative clustering. The measure of dissimilarity defines clusters that will be combined in the case of an agglomerative method, where we use the Ward method as linkage method for calculating the inter-cluster distances. Dendrograms progressively show how the number of small-sized low hierarchy clusters increases over time due to the higher nodes switching in terms of strategies. This is due to change in cooperativeness weighted by the happiness index, eventually impacting on diets and gut bacterial composition. In other words, the increasing number of small groups reflects the growing presence of newborn clusters composed by only a small number of nodes with a high similarity in gut bacterial composition.

**Fig 4:**
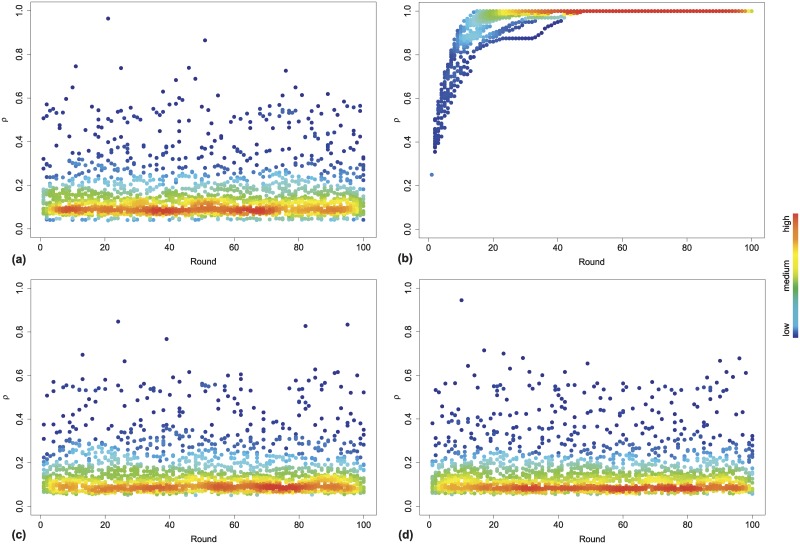
Evolutionary dynamics of the density of cooperative nodes over time. We show the fraction of cooperative nodes *ρ*_*i*_ against the rounds or time steps (*t*) before feedback (a) and after the first feedback round (b), the second feedback round (c) and the third feedback round (d). *ρ* ranges in [0, 1], where 0 corresponds to the global defection, while 1 means a global cooperation of population. We simulated the evolutionary dynamics of the ISG for a fixed number of simulations, and the color, ranging from ‘blue’ to ‘red’, corresponds to the density *ρ*_*i*_. We can observe a fluctuation in strategies determined by the feedback rounds from gut acting alternatively as incentives towards cooperativeness (see (b)) and a coexistence of the two strategies, with more defectors than cooperators (see (a), (c) and (d)).

Fig 4 shows the evolution of cooperation, namely the density of cooperative nodes against the rounds. We simulated the evolutionary dynamics before feedback from gut and after each feedback round, for a number of rounds such that a dynamical steady-state was reached. Before feedback we can observe the initial coexistence of both strategies with a presence of more defectors than cooperators, as expected [60]. After the first feedback round, an overall tendency to cooperate emerges, so that the density of cooperators reaches the maximum value. This is the result of the immediate reaction of individuals driven by the feedback from their gut microbiota. This first feedback round acts as an initial incentive to cooperate. The reciprocity effect amplifies this incentive, therefore leading to an overall cooperativeness in the multiplex network. Then, the second feedback round produces a fluctuation in strategies with a coexistence of both defection and cooperation, with defectors outnumbering cooperators.

The third feedback round confirms what we observed in the second feedback round, with a similar fluctuation in strategies. Overall, happiness index, gut bias and gut similarity play a major role in shaping behaviors, since they lead to strong fluctuations over time and heterogeneous reactions of nodes, also due to the multiplex nature of interactions. The observed fluctuations in strategies, with an overall prevalence of defectors, is what we expected from the case of shift-working, and reflect the social dynamics of chrono-nutrition. In fact, shift-working, the altered lifestyle and circadian rhythm and consequently the impact on their gut microbiota (connected to the cellular metabolism) [57, 88], along with the interplay with human behavior, mainly leads to dysregulated feeding habits. Lack of sleep and changes to the life cycle induce shift workers to choose snacks or fast high-fat meals (i.e., defection), giving them a false sense of energy and a sort of illusory satisfaction as a consequence [36, 38, 41, 54]. In this way, they try to counterbalance their stress (micro-inequity) by choosing high-sugar foods and producing a sort of micro-affirmation, regardless of what the social and microbial communities may try to induce.

Thus, psychological biases and the interplay with gut microbiota, statistically quantified by happiness index and gut bias, shape the evolutionary outcomes. The forced oscillations or fluctuations observed in the case of shift-working are due to the fact that the altered feeding habits, owing to shifting, are quicker than gut microbial adaptation. This can be associated to a form of hysteresis in the formation of adapted microbial communities, whose variability is slower than shifting. In other words, this finding seems to confirm the crucial role of meal timing, and how the dynamic interplay in the case of shift-working produces poor food choices and a higher energy intake [41]. Furthermore, the feedback mechanisms from the gut, social and microbial communities are not able to counterbalance this tendency to defect.

Although, in some temporal windows, multiplexity can drive nodes to replicate strategies through layers by amplifying their effect, sometimes it acts in the opposite direction, averaging its impact in the two layers of interaction. To unveil the role of homophily, in S1 Fig we show the evolutionary dynamics considering the two cases of high (*σ* = 1) and low homophily values (*σ* = 8). As expected from the findings of [30], the figure highlights how the higher is the homophily value, the more nodes tend to cooperate.

### Correlation analysis

To gain insight into how the “happiness index” is influenced by particular pathways, we here investigate the correlation of *γ*_*i*_ with absolute average flux rates across subsystems in the model. We observe correlations between average flux in each pathway and happiness index in each node, or diet. Our table of results for the correlation analysis is provided in S1 Table. Overall, the positive correlations which stand out as being significantly higher are folate metabolism and purine/pyrimidine synthesis in the pathogen *S. typhimurium*, during the second feedback round (> 0.5). These pathways are linked in several ways, e.g. the folate cofactor tetrafolate is involved in the synthesis of purines [89].

Both these pathways are also responsible for producing fundamental biomass precursors, which is significant as biomass is the objective maximized for all species in our model during FBA. It is well established that purines and pyrimidines form the building blocks for the nucleotides which comprise DNA, but they are also vital for the synthesis of important coenzymes, such as ATP and NAD. Folate is required for important cellular processes such as DNA replication, repair and methylation, as well as playing a vital role in the metabolism of many amino acids. Maternal diet, such as periconceptional folic acid supplementation, has proven to impact DNA methylation in offspring and can affect their neurodevelopment and health outcomes [90]. In humans, folate cannot be synthesized *de-novo* and must therefore be obtained from the diet or gut microbiota. In this regard, the increase in dietary folate intake and introduction of folate-producing probiotic bacteria in the form of live microbial supplements was previously discussed [91].

In accordance with protein-rich diets yielding a high *γ*_*i*_ value, it can be seen that the metabolism of various amino acids are among the top positively and negatively correlated subsystems with *γ*_*i*_ in all four feedback rounds. In the first and second feedback rounds, the synthesis and recycling of murein in *E.coli* has a significant role to play, as murein is a polysaccharide which provides structural integrity to bacterial cell walls whilst allowing the exchange of metabolites [92, 93].

As a key intermediate in human cellular respiration, acetyl co-enzyme A is involved in pyruvate oxidation, as well as cholesterol synthesis and fatty acid oxidation during lipid catabolism, where it acts as an acyl carrier [94]. Fatty acids are an essential component of both human and bacterial cell membranes and can be synthesized via glycolysis or amino acid deamination. Human cholesterol metabolism (in the third flux iteration) has a moderately high negative correlation; this seems to support the idea that diets high in cholesterol are likely to lead to poor health.

## Discussion

Our work has investigated and modeled the “gut-human behavior axis” in order to quantify the bidirectional interplay between social behaviors, dietary habits and the gut microbiota. To this end, we have analyzed the evolutionary dynamics of dietary behaviors using a realistic social dilemma (i.e., the snowdrift game), in a real-world scenario represented by the social multiplex network. We have included a dual concept of similarity, namely homophily and gut similarity. We have further considered psychological biases, unveiling how and to which extent they are able to modulate human food choices and gut bacterial population. To quantify the “gut-human behavior axis”, we have introduced a feedback mechanism, acting from dietary behaviors in the social multiplex network to the gut microbiota, and vice-versa. In particular, we have focused on the social dynamics modeling of chrono-nutrition in the case of shift-working, in order to model and understand the impact of the interplay between human behavior and gut microbiota on the feeding habits of shift workers and on their gut microbiota.

Our aim was to shed light on the role of timing in this interplay with their gut, along with the impact of social multiplex environment and interactions on feeding habits. We have therefore introduced statistical estimators able to quantify the relation between human behaviors and gut microbiota in a social multiplex network, exploring its role in influencing dietary behaviors. Although the immediate reaction of individuals driven by the first feedback round from gut is an overall emergence of cooperation, we subsequently observed a fluctuation in strategies, with a coexistence of the two strategies and more defectors than cooperators. “Happiness index”, “gut bias” and “gut similarity” control the gut microbial communities and social associations, shaping human behaviors. This leads to strong fluctuations over time and heterogeneous reactions of nodes in the same temporal window, due to the multiplex nature of interactions. The multiplexity of the networked nodes, sharing a similar diet and gut microbial composition, leads to the formation of both cooperative and defective groups, evolving over time according to the re-clustering due to the feedback rounds from gut.

Thus, the game-theoretic approach has allowed us to obtain a multi-scale integration of different aspects, ranging from complex networks to metabolic issues, other than including psychological and cognitive biases, in the investigation and modeling of the social dynamics of chrono-nutrition. We envisage the use of such network approaches coupled with GSMMs to investigate the relationship among individuals (or dietary patterns) in a network layer, as well as the relationship between the various omics, which can be modeled as individual layers of a multi-layer network [35]. Additionally, by correlating diets with “happiness” there is a possibility to identify dietary behaviors which are beneficial or detrimental to human health. The proposed model could represent a class of models that may be adapted and refined along with the growing amount of information on the gut environment and the gut-brain axis. Moreover, what further emerges is how the impact of both the “gut-human behavior axis” and the gut microbiota-brain axis could be even more important to be studied in children with the potential to prevent the development of abnormal behaviors or disorders [95] and opening novel paths for nutritional or therapeutic interventions in at-risk population.

Our work opens the path for a deeper analysis on the impact of feeding behaviors, such as in the case of shift workers, on the resulting metabolic issues and diseases. In fact, to deepen our understanding on the dynamics of feeding habits, as well as taking into account a wide variety of aspects, including educational and familiar habits, geographical location, psychological factors (e.g. mood, stress, impulsiveness), physiological conditions [6, 21] and inherited factors, we also need to consider social influences and similarities. To this end, we have introduced gut similarity and homophily between individuals, and we have quantified the dynamic interplay between human behavior and gut microbial population, considering the multiple aspects of the social environment (workplace, friends, etc.) where shift workers are included and interact with each other. Modeling and quantifying the social dynamics of chrono-nutrition may allow the different actors (physicians, psychologists, educators, legislators, etc.) to influence and modify shift workers’ eating habits with targeted intervention strategies acting on focus groups (e.g. by introducing food-sharing in the workplace) [49], pushing individuals towards cooperation, based on incentives and mechanisms of direct and indirect reciprocity [62]. A further approach could be increasing their overall awareness on dietary behaviors, in terms of quality, quantity and timing of meals, highlighting possible consequences on health.

Given the rise in nutritional studies that address the effect of timing in feeding behaviors on obesity and metabolic syndrome [58], along with the increasing importance of gut microbiota into the studies related to circadian rhythms [37, 57, 96, 97], this novel social multiplex perspective on the dynamics of chrono-nutrition enriched by psychological and social statistical estimators may pave the way to gain a better understanding on the impact of social multiplex dynamics on the interplay between human feeding behaviors and gut microbiota. This can provide new insights on the clock-nutrition pathogenesis of several diseases of the digestive system, and can suggest key strategies toward targeted therapeutic interventions for metabolic diseases and other pathologies [98].

### Code availability

The developed methodology in terms of metabolic modeling, that is the multi-objective optimization of gut community metabolic model, is available to the scientific community at the following repository: https://github.com/svijayakumar32/multi-objective-optimization-of-gut-community-metabolic-model.

## Supporting information

S1 Fig The role of homophily in the evolutionary dynamics of the density of cooperative nodes over time. We show the evolutionary dynamics considering the two cases of high (*σ* = 1)(a) and low homophily values (*σ* = 8)(b) (where *σ* is the standard deviation of the normal distribution). Plots highlight how a high homophily value encourages cooperation in the multiplex network.
(EPS)

S1 Table Correlation analysis. Top 10 positive and negative Pearson correlations calculated between average subsystem fluxes and at each feedback round.
(PDF)
