# Supplementary material for: Social dynamics modeling of chrono-nutrition
Source: PLoS Comput Biol. 2019 Jan 30;15(1):e1006714. doi: 10.1371/journal.pcbi.1006714 (PMC6370249; doi:10.1371/journal.pcbi.1006714)
Supplement: S1 Table — Top 10 positive and negative Pearson correlations calculated between average subsystem fluxes and at each feedback round. (PDF) [file pcbi.1006714.s002.pdf]

Feedback One	
**Top 10 Positively Correlated Fluxes**	
**Subsystem**	**Correlation**
'KP' "Tyrosine metabolism"	0.37807811
'EC' "Murein Biosynthesis"	0.35593737
'ECs' "Murein Recycling"	0.35549066
'ECs' "Murein Biosynthesis"	0.35531688
'ECs' "Threonine and Lysine Metabolism"	0.26658649
'ECs' "Glycine and Serine Metabolism"	0.26654447
'ECe' "Tyrosine Tryptophan and Phenylalanine Metabolism"	0.25188833
'STy' "Threonine and Lysine Metabolism"	0.24643722
'STy' "4 hydroxyphenylacetate catabolism"	0.23957028
'FP' "Unassigned"	0.22898343
**Top 10 Negatively Correlated Fluxes**	
**Subsystem**	**Correlation**
'BT' "Unassigned"	-0.2324472
'ECs' "Oxidative Phosphorylation"	-0.2197121
'LL' "Valine Leucine and Isoleucine Metabolism"	-0.217536
'HS' "Fatty Acid Synthesis"	-0.2046681
'FP' "Arginine and Proline Metabolism"	-0.1842717
'LP' "Alternate Carbon Metabolism"	-0.1819074
'STy' "Transport"	-0.1813692
'ECs' "Pyruvate Metabolism"	-0.1669255
'KP' "Butanoate Metabolism"	-0.1645353
'KP' "Oxidative Phosphorylation"	-0.1603565
**Feedback Two**	
**Top 10 Positively Correlated Fluxes**	
**Subsystem**	**Correlation**
'STy' "Folate Metabolism"	0.59036697
'STy' "Purine and Pyrimidine Biosynthesis"	0.56859907
'STy' "Glycine and Serine Metabolism"	0.30534423
'EC' "Murein Biosynthesis"	0.28610424
'HS' "Heme degradation"	0.26066047
'BT' "Folate Metabolism"	0.2356027
'ECs' "Murein Recycling"	0.22444353

'STy' "Glyoxylate Metabolism"	0.2131902
'HP' "ROS Detoxification"	0.20816944
'HS' "Arginine and Proline Metabolism"	0.2055416
**Top 10 Negatively Correlated Fluxes**	
**Subsystem**	**Correlation**
'HP' "dUTP Biosynthesis"	-0.2219613
'ECe' "Membrane Lipid Metabolism"	-0.2138996
'ECs' "Histidine Metabolism"	-0.20623
'ECs' "Citric Acid Cycle"	-0.1960893
'STy' "Exchange"	-0.1955515
'HP' "dTTP Biosynthesis"	-0.1829439
'EC' "Citric Acid Cycle"	-0.1774323
'ECe' "Inorganic Ion Transport and Metabolism"	-0.1770236
'ECs' "Alternate Carbon Metabolism"	-0.1741285
'HP' "Glycerolipid Synthesis"	-0.1558785
**Feedback Three**	
**Top 10 Positively Correlated Fluxes**	
**Subsystem**	**Correlation**
'LL' "Pentose Phosphate Cycle"	0.3745564
'LL' "Tyrosine Tryptophan and Phenylalanine Metabolism"	0.36899784
'EC' "Cysteine Metabolism"	0.32391016
'HS' "CoA catabolism"	0.32132078
'STy' "Methionine Metabolism"	0.30245529
'KP' "Riboflavin Metabolism"	0.28484411
'LL' "Lipid Cell Wall Metabolism"	0.20341677
'ECs' "Unassigned"	0.19387464
'ECs' "Glutamate Metabolism"	0.18582804
'LL' "Alternate Carbon Metabolism"	0.18162498
**Top 10 Negatively Correlated Fluxes**	
**Subsystem**	**Correlation**
'HS' "Cholesterol metabolism"	-0.32022295
'HS' "Aminosugar Metabolism"	-0.28879565
'BT' "Glycine, Serine and Threonine Metabolism"	-0.20810029
'BT' "Central Metabolism"	-0.19594971
'HS' "Transport, intercellular"	-0.19568761

'ECs' "Glycine and Serine Metabolism"	-0.18777028
'ECs' "Threonine and Lysine Metabolism"	-0.18035997
'HS' "C5-branched dibasic acid metabolism"	-0.17974225
'ECs' "Pyruvate Metabolism"	-0.17746382
'LL' "Methionine Metabolism"	-0.17189016
**Feedback Four**	
**Top 10 Positively Correlated Fluxes**	
**Subsystem**	**Correlation**
'EC' "Nitrogen Metabolism"	0.418806615
'ECs' "Glycerophospholipid Metabolism"	0.387722707
'ECe' "Glycerophospholipid Metabolism"	0.305090808
'KP' "Benzoate Degradation"	0.248625539
'KP' "Nitrogen Metabolism"	0.248442119
'KP' "Phenylalanine Metabolism"	0.246854193
'STy' "Anaplerotic Reactions"	0.226538326
'LL' "Cofactor and Prosthetic Group Biosynthesis"	0.216393231
'HS' "ROS Detoxification"	0.20917127
'ECe' "Threonine and Lysine Metabolism"	0.20755859
**Top 10 Negatively Correlated Fluxes**	
**Subsystem**	**Correlation**
'LL' "Arginine and Proline Metabolism"	-0.29882075
'KP' "Cell Envelope Biosynthesis"	-0.2401224
'ECs' "Tyrosine Tryptophan and Phenylalanine Metabolism"	-0.22149735
'KP' "Transport, Extracellular"	-0.21482511
'HS' "Transport, peroxisomal"	-0.20553729
'KP' "Transport Outer Membrane"	-0.19956758
'LL' "Valine Leucine and Isoleucine Metabolism"	-0.19853544
'EC' "Glycolysis/Gluconeogenesis"	-0.19484854
'EC' "Alternate Carbon Metabolism"	-0.19214115
'LL' "Central Metabolism"	-0.18940579
